# Case of Epileptic Mania

**Published:** 1883-08

**Authors:** John C. Robert

**Affiliations:** New Utrecht, New York


### CLINICAL BUREAU.

### CASE OF EPILEPTIC MANIA.

JOHN C. ROBERT, NEW UTRECHT, NEW YORK.

May 15th, 1882.—I was called to visit a medium-sized lady, with dark-blue eyes, dark-brown hair, moderately stout, intellectual faculties good, lively and cheerful disposition, and 28 (twenty-eight) years of age, who has recently become insane. Her insanity is supposed by her friends to have resulted from a fright, occasioned by a fire which took place in the house a month ago about 6 o'clock P. M.

After the fire was extinguished, she was found lying unconscious in the back yard, and after she was taken into the house she had a succession of epiletic fits, which continued for two days. In answer to my questions, I was informed that two weeks before the fire she lost her only child, about eighteen months old, after six weeks of care and anxiety.

During her first confinement, eight years ago, she had convulsions, for which her *homœopathic* physician gave her chloroform. She was under its influence two hours without effect, the convulsions not ceasing until the child was born.

During the first week of her insanity, her disease was of the following regular type:

1st. Epileptic convulsions coming on regularly twice a day, between 3 and 4 o'clock, P. M. and between 3 and 4 o'clock, A. M. A very fetid breath preceded the convulsions, which lasted about ten or fifteen minutes, and terminated in unconsciousness.

2d. Soon after she became conscious, maniacal delirium began, accompanied with severe spasms of the limbs and face, and lasting half an hour or more, and in which she is possessed of great strength and is very violent and tries to escape from bed and get out of the house, and tries to strike and injure those around her. She wants the carving-knife to stab her favorite sister, and she sometimes howls like a dog.

This stage is shorter or longer, according as her attendants seize her promptly or not, and consists of a succession of fits of delirium, lasting three or four minutes, with intervals of repose of about eight to ten minutes.

3d. Stage of exhaustion, in which she sleeps for an hour or more. When she awakens from this sleep, she is very weak and helpless and can neither feed herself nor turn herself in bed.

During this stage, at night, she is sleepless, and talks incessantly of the different objects of her hallucinations, but her mind can be diverted from these hallucinations by asking her questions about her symptoms and questions about her desires for food or drink and other questions respecting her comfort, all of which she answers correctly, but speaks slowly, much more slowly than is her natural habit. In the daytime she is drowsy and stupid, talking very little.

Since the commencement of her illness, she has been under allopathic treatment, which has had no other effect than to aggravate and alter the type of the disease and make it irregular, the paroxysms coming on at different hours and continuing longer.

The physicians in charge of her decided yesterday that she must be sent to a lunatic asylum, a step to which her family are opposed. After seeing the patient and expressing the opinion that she should not be sent to an asylum and that she could be much better treated at home, I was at once requested to take charge of her. As she was under the influence of Morphine, I did not attempt to talk to her, but gave her one powder of Bell.$^{cm}$ (Fincke) dry, and left one powder to be dissolved in one-third of a tumbler of water and a tablespoonful to be given every two hours.

In the course of a few visits, I obtained the following symptoms: She has many hallucinations; she sees demons in the room; she sees a man looking through what she imagines to be a hole in the wall and who has come to carry her off; she sees strange men lurking about who intend some mischief; she says that she was murdered last night, and that her hands were cut off. The chandelier at the foot of the bed troubles her greatly; she imagines that some one has lighted the gas and is melting lead in the chandelier, which runs down upon her and burns her dreadfully; she says this is not her house, and that her house was burned down last night and that an explosion took place which blew it to pieces. She complains that her friends carry her about from house to house.

She keeps her eyes closed most of the time, and when they are open she cannot see, the pupils being very much dilated. She cannot bear the light.

She has severe stitching pain in upper part of forehead; dull pain in lower part of forehead over nose; stitching pain over eyes; pain in vertex, as from a weight; throbbing pain in temples; her forehead feels as though grasped by a band of iron, and she has her head bandaged to allay the pain. She does not urinate as frequently as she should, sometimes only once in forty-eight hours; the urination is preceded by pain in the hypogastrium. This has been her habit for a long time whenever she is sick, and at other times she is not as regular as she should be.

She complains of a feeling as from a lump in the pit of the stomach; hiccough, involuntary diarrhœa. For some time past her menses have come on twice a month. Appetite poor; some days eating nothing.

May 16th.—Saw her this morning and think that there is a slight improvement, but her attendants say that she is much worse and that she had a very bad night and gave them a great deal of trouble. I thought possibly Bell.$^{cm}$ (Fincke) had aggravated her symptoms and gave Bell.$^{m}$ (Fincke), one powder at night and one in the morning, and giving her one myself before I left the house.

May 17th.—This morning I find her much better. Her attendants say that she passed a very good night and that she had neither convulsions nor delirium. Her hallucinations still continue, but she is not violent. Gave Bell.$^{m}$ (Fincke) dry, night and morning.

May 18th.—Better. Her headache is better and the bandage that was around her head has been taken off, and her friends begin to entertain hopes of her recovery. Gave Bell.$^{m}$ (Fincke) dry, night and morning.

May 19th.—Not so drowsy; keeps her eyes partly open. I am able to hold a long conversation with her, and find that her hallucinations are beginning to disappear. She complains still of throbbing pains in the temples; pain across the forehead as if from a band of iron; dull pain over the root of the nose; stitching pains over the eyes, and has some difficulty in keeping her eyes open. The pain in vertex is much better. Gave Bell.$^{m}$ (Fincke) dry, night and morning.

May 20th.—The pain in vertex has left her, but she still complains of a pain as from a band of iron across her forehead, and that everything appears green before her eyes. Her hallucinations still continue but are gradually disappearing. She lies quietly in bed and is perfectly harmless and does not seek to escape. She is very weak and exhausted; no pulse to be felt. Gave Bell.$^{m}$ (Fincke) dry, night and morning.

May 23d.—Talks more rationally to-day; objects do not appear green, as formerly. Is quite pleasant and cheerful and converses on several subjects. She is still very weak, and still complains of pain as from a band of iron across her forehead, and of the throbbing pain in her temples, but says that these pains are not so bad as they were, and the pain in temples not so constant. She has some pain in occiput and complains of vertigo when her attendants attempt to raise her head from the pillow. Gave Bell.$^{m}$ (Fincke) night and morning.

May 24th.—Worse to-day; talks of fire; says that people are trying to shoot her; complains of a lump in pit of stomach; dull pain in forehead over root of nose and over the eyes; no appetite, has eaten nothing to-day. She is quite cross. Gave Nux vom.$^{m}$ (Fincke) night and morning.

May 25th.—Better to-day; appetite better; desires better food, something more substantial. The pain over the eyes and nose has left her. No sensation as from a lump in pit of stomach. She has a slight pain in upper part of forehead; pain in temples; soreness of scalp above temples; vision is improving. Gave Nux vom.$^{m}$ (Fincke) night and morning.

May 26th.—Improving. All her hallucinations have entirely disappeared. Slight headache. Gave Nux vom.$^{m}$ (Fincke) night and morning.

May 27th.—Difficulty of urination, preceded by pain in hypogastrium; headache much worse—says it aches so that it almost sets her crazy. Gave Canth.$^{cm}$ (Fincke), a powder dissolved in one-third of a tumbler of water, and a tablespoonful to be given every hour.

5 P. M.—Called again to-day at 5 P. M., and found that she was relieved of the difficulty of urination and pain in the hypogastrium, and passed water soon after taking the first dose. Says she feels well, excepting a slight pain in upper part of forehead. Appetite good; pulse regular, but small; feels very weak; pupils normal. Continued Canth.$^{cm}$ (Fincke) in water.

May 28th.—She had a slight return of convulsions this morning, but she kept control of herself. No trouble with urination. Had slight stitching pain in upper part of forehead. Had throbbing pain in temples this morning, which was relieved by the nurse pressing the temples firmly with both hands. She is sullen and taciturn; heaviness of eyes; drowsy; no sleep last night; no appetite; feels weak and exhausted. Gave Nux vom.$^{m}$ (Fincke) dry, night and morning.

May 29th.—Much better to-day. Slept well last night; appetite good; complains of pain in os coccygis; has a feeling of coldness running down her spine. (She had this coldness running down her spine a month before her illness, and felt very cold all last winter.) When she is raised up in bed by the nurse everything turns green before her. Her lower limbs are partially paralyzed; she has very little power over them; she cannot stand on them, but only move a little in bed. She can move her arms and hands, but with difficulty, and when I ask her to raise her hands to her head her arms move slowly and very unsteadily, and the fingers are stiff. Gave Rhus tox.$^{m}$ (Fincke) night and morning.

May 30th.—No pain in os coccygis; feels very well; has better use of her hands; fingers are yet stiff; lower limbs slightly improved; urination continues regular. Continued Rhus tox.$^{m}$ (Fincke) night and morning.

May 31st.—Sharp pains through temples; soreness of scalp at vertex; sharp pains through forehead; slept well last night. Continued Rhus tox.$^{m}$ (Fincke) night and morning.

June 1st.—Vertex sore. All other symptoms have disappeared excepting a slight stiffness of fingers and the partially paralyzed condition of the lower limbs. Mind perfectly clear. No urination to-day. Continued Rhus tox.$^{m}$ (Fincke) night and morning.

June 2d.—Slept well last night. She awakened this morning at 8 o'clock and complained of confusion of mind, and shortly after had convulsions for about half an hour; sometime after complained of pain in hypogastrium and about 11 A. M. urinated, passing a quart of highly colored urine. Vertex sore only when touched; more power in lower limbs; appetite good. Gave Bell.$^{m}$ (Fincke) night and morning.

June 3d.—Urinated this morning without pain. Had headache in forehead for about half an hour this morning, and dull pain in vertex; not so much confusion of mind; some vertigo when she is raised in bed. Bell.$^{m}$ (Fincke) night and morning.

June 4th.—Has not urinated this morning. Had headache for about three hours last night, commencing at the middle of forehead and going around on both sides to back of head. She is now able to feed herself, which she could not do before on account of the stiffness and inability to bend her fingers and the want of power in her arms and hands. She complains of a lump in pit of stomach, nausea, and fetid breath. Nux vom.$^{m}$ (Fincke) night and morning.

June 6th.—Much worse to-day. Had convulsions this morning, lasting half an hour. Before the convulsions breath smelt fetid, and she perceived a gluey taste in the mouth. After the convulsions, she urinated without pain. Vertigo whilst lying in bed. Bell.$^{m}$ (Fincke) night and morning.

June 7th.—Pain when urinating, but this is in the urethra. Slept well last night. Bell.$^{m}$ (Fincke) night and morning.

June 8th, 5½ P. M.—Convulsions at 5 P. M., lasting fifteen minutes —had headache and fetid breath before the attack. After the attack she fell asleep, and awakened at the sound of my voice a few minutes after I entered the room. Appetite good; slept well last night; mind clear. Nux vom.$^{m}$ (Fincke) night and morning.

June 9th, 5½ P. M.—No convulsions to-day. Feels stronger and is able to walk about the room with assistance. Appetite good; slept well last night; makes no complaint. As Bell. and Nux do not act to my entire satisfaction, I begin to think of Psora, although I find no history of it, and gave Sulph.$^{m}$ (Fincke) night and morning.

June 10th, 5½ P. M.—About half an hour ago she complained of a gluey taste in the mouth (this gluey taste always precedes an attack of convulsions). She then fell asleep for about a quarter of an hour. She says now that she feels perfectly well, but weak. Face covered with pimples. Continued Sulph.$^{m}$ (Fincke) night and morning.

June 17th.—Sulph.$^{m}$ (Fincke) was continued until to-day, and under which she has improved very rapidly. This morning she had a light fit about 11 o'clock; this fit was preceded by a gluey taste in the mouth, but no fetor of breath. She does not complain of anything except pain in vertex and weakness. Bell.$^{m}$ (Fincke) night and morning.

June 18th.—Had headache to-day. Is still very weak. No convulsions. Gave Arsen. album.$^{m}$ (Fincke) night and morning.

June 30th.—She has taken Ars. a.$^{m}$ (Fincke) every day, and has improved very rapidly under it. She is able to go about the house without assistance, with the exception of going up and down stairs, which she cannot do without assistance, as her knees are yet too weak. She had a light fit this morning. Gave Calc. carb.$^{m}$ (Fincke) night and morning.

July 1st.—Throbbing in vertex. No other symptoms. Continued Calc. carb.$^{m}$ (Fincke), night and morning.

July 14th.—Calc. carb.$^{m}$ (Fincke) has been continued every day, night and morning, and she now feels perfectly well. Her knees are still weak, and going up and down stairs causes pain in them; this is probably due to tenderness at the insertion of muscles at the knees. Gave Symphytum$^{cm}$ (Fincke), night and morning.

July 17th.—Symphytum has been given every day, and now she can go up and down stairs without the slightest pain. She complains of a slight gluey taste in the mouth. Gave Calc. carb.$^{m}$ (Fincke) night and morning.

Aug. 20th.—Calc. carb.$^{m}$ (Fincke) has been given every day up to the 29th of July, on which day she left home on a visit to the Catskill Mountains in perfect health. To-day I saw her, and find that there has been no return of the former symptoms. Gave nothing.

Sept. 9th.—A few days ago she had an attack of diarrhœa, brought on by eating excessively of raw tomatoes, and being desirous of going to a picnic, she took a few large doses of paregoric. Yesterday she had a slight attack of convulsions, which was promptly subdued by Bell.$^{cm}$ (Fincke) given according to the directions which I had left with her friends. She is very despondent to-day. Gave Ignatia$^{m}$ (Fincke) night and morning.

Sept. 28th.—Epileptic fit, preceded by suppression of urine for twenty-four hours. Gave Bell.$^{cm}$ (Fincke).

June 7th, 1883.—She has not had any return of the epileptic fits since September 28th.

I have treated her through the winter for colds, suppression of urine, and headaches, and since the middle of March she has been perfectly well, and has recovered all the former buoyancy of spirits usual to her before marriage.

All her friends say that she has not been so well in ten years.

This case of insanity was treated entirely upon the very strictest of homœopathic principles, and no advantage was taken of the new code to consult with any physician of the old school, or of any other school. The family of the patient desired to have the homœopathic treatment, and no attempt was made to practice fraud on them by pretending that it was one of those special cases which required the allopathic practice, wholly or in part.

The allopathic treatment consisted of Bromide of Potassium, Opium, Chloral, Morphine, and a number of other remedies in combination. Both of her children died during dentition under the allopathic treatment.

This case proves, without controversy, that the large doses employed only brought the patient to the last extremity of furious mania, which, aided by the help which the old school can afford, would surely have resulted in death, and that even under such trying circumstances the infinitely small doses of modern Homœopathy have been successful in leading the case to the desired end of curing it. Nay, it shows conclusively that when the organism is in such a highly excited state, nothing will more favorably reach it than the Hahnemannian infinitesimals, so-called, and that nothing is so calculated to increase the danger and bring about the undesired end, which is death under most aggravating forms, than the great palliatives of the old school.

